# Cardiovascular Implications of Intermittent Hypoxia: A Comprehensive Narrative Review

**DOI:** 10.7759/cureus.97121

**Published:** 2025-11-17

**Authors:** Gayathri Dantu, Sai Venkata Siddhartha Masetti, Tanvi Barsinge, Alekhya Madarapu, Siddharth Ragupathi, Malika Inoyatova, Chaitanya Kumar Javvaji

**Affiliations:** 1 Internal Medicine, Government Medical College and Hospital, Mahabubnagar, IND; 2 Medicine, Bustamante Hospital for Children, Kingston, JAM; 3 Internal Medicine, Seth Gordhandas Sunderdas Medical College and King Edward Memorial (KEM) Hospital, Mumbai, IND; 4 Internal Medicine, Government Medical College and Hospital, Nizamabad, IND; 5 Internal Medicine, Saint Peter’s University Hospital, New Brunswick, USA; 6 Internal Medicine, Memorial Healthcare System, Pembroke Pines, USA; 7 Pediatrics, Jawaharlal Nehru Medical College, Datta Meghe Institute of Higher Education and Research, Wardha, IND

**Keywords:** arrhythmias, cardiovascular disease, heart failure, hypertension knowledge, intermittent hypoxia (ih), oxidative stress

## Abstract

Intermittent hypoxia (IH), particularly in obstructive sleep apnea (OSA), has a complex role in cardiovascular health, acting both as a risk factor for disease and, under specific circumstances, as a therapeutic tool. This narrative review aims to explore the molecular mechanisms by which IH contributes to cardiovascular diseases, including hypertension, heart failure, arrhythmias, and atherosclerosis. Key processes such as oxidative stress, sympathetic hyperactivity, and hypoxia-inducible factor 1-alpha (HIF-1α)-mediated inflammation are discussed in relation to vascular and myocardial remodeling. Clinical and experimental data highlight the significant association between IH and increased cardiovascular morbidity and mortality, especially in high-risk populations. This review also evaluates the effectiveness of treatments like continuous positive airway pressure (CPAP), mandibular advancement devices, weight reduction, pharmacotherapy, and intermittent hypoxic conditioning (IHC). It emphasizes the importance of early screening and personalized treatment strategies for better patient outcomes. Emerging research on IH-based cardioprotection and biomarker discovery suggests promising avenues for future therapies. Ultimately, IH has both detrimental and potentially beneficial effects on cardiovascular health, depending on its context and management.

## Introduction and background

In recent years, intermittent hypoxia (IH) has been recognized as a significant factor affecting cardiovascular health. Chronic IH is a term used to describe a physiological or pathological pattern of oxygen dysregulation that is characterized by frequent, brief (seconds to minutes) cycles between low and normal physiological levels of oxygen, often occurring many times per hour [1]. It has been demonstrated that controlled exposure to IH has positive effects on the cardiovascular system, including lower systolic and diastolic blood pressure, which are mostly attributable to enhanced vascular and autonomic function [2,3]. However, the effects of IH are highly dose dependent. While mild, regulated IH combined with exercise can induce beneficial cardiovascular adaptations, such as enhanced vascular function, increased maximal oxygen uptake (VO₂max), and improved overall cardiovascular performance primarily through the activation of hypoxia-inducible factor 1-alpha (HIF-1α)-mediated pathways, severe or prolonged hypoxia such as that seen in obstructive sleep apnea (OSA), can contribute to pathological processes like atherosclerosis, vascular remodeling, and heart failure, all of which share tissue hypoxia as a common feature [2-4]. Through a variety of processes, such as recurring cycles of oxygen deprivation and reoxygenation that produce oxidative stress and reactive oxygen species (ROS), which in turn cause endothelial damage, IH contributes to cardiovascular dysfunction. Vasoconstriction and chronic hypertension are induced by this oxidative stress when combined with sympathetic nervous system activation. In addition, IH causes systemic inflammation that is marked by increased cytokine levels, including tumor necrosis factor-alpha (TNF-α) and interleukin-6 (IL-6), which worsens endothelial function and accelerates atherosclerosis [5].

IH is experienced in a variety of clinical and experimental settings. Pathologically, IH is most commonly seen in situations including heart failure, chronic obstructive pulmonary disease (COPD), central sleep apnea, OSA, and preterm infants with immature respiratory control [5]. IH commonly occurs during high-altitude exposure, and experimental models replicate similar conditions seen in deep-sea diving and high-intensity hypoxic training, which are used to study its physiological effects and potential therapeutic applications. The adaptive responses to hypoxia are largely mediated by the activation of HIF-1α, which upregulates genes involved in angiogenesis (vascular endothelial growth factor-a, or VEGF-a), erythropoiesis (erythropoietin, or EPO), cellular metabolism (pyruvate dehydrogenase kinase isoform 1, or PDK1; lactate dehydrogenase-a, or LDH-a), and inflammation (inducible nitric oxide synthase, or iNOS) [6-10]. Recent data highlights the complex metabolic reprogramming brought on by IH, which is mostly mediated by HIF-1α activity. PDK1 is upregulated in one important mechanism, which inhibits pyruvate dehydrogenase and causes cellular metabolism to shift toward anaerobic glycolysis [9]. By lowering mitochondrial oxygen consumption, this metabolic switch allows cells to produce ATP while adapting to low-oxygen conditions.

**Figure 1:**
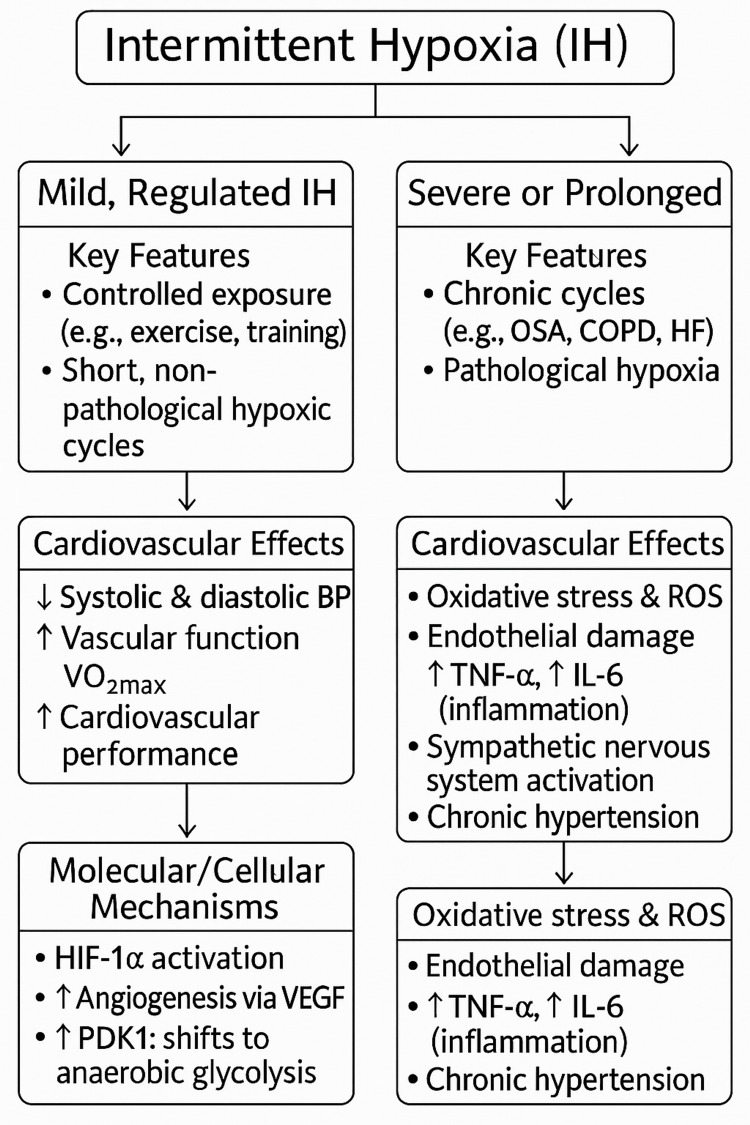
A schematic figure summarizing the dual role of intermittent hypoxia in cardiovascular physiology IH: intermittent hypoxia; OSA: obstructive sleep apnea; HF: heart failure; COPD: chronic obstructive pulmonary disease; BP: blood pressure; VO_2max_: maximal oxygen uptake; HIF-1α: hypoxia-inducible factor 1-alpha; VEGF: vascular endothelial growth factor; PDK1: pyruvate dehydrogenase kinase isoform 1; ROS: reactive oxygen species; TNF-α: tumor necrosis factor-alpha; IL-6: interleukin-6 Image created by Sai Venkata Siddhartha Masetti

Simultaneously, IH promotes the development of monocarboxylate transporters (MCT1 and MCT4), which help hypoxic tissues better regulate pH and facilitate lactate export [8]. Chronic IH also promotes capillary density and tissue perfusion by upregulating VEGF and its receptors kinase insert domain receptor (KDR/Flk-1) and Fms-like tyrosine kinase-1 (Flt-1), which increases angiogenic capacity [10]. Figure 1 presents a summary of the role of intermittent hypoxia as discussed.

Given the complexity of IH’s effects on cardiovascular physiology, the objectives of this review are to provide a comprehensive analysis of the role of hypoxia in cardiovascular health and to clarify the pathophysiological mechanisms that underlie the development and progression of cardiovascular diseases (CVDs) like ischemic heart disease, heart failure, and pulmonary hypertension (PH) by influencing myocardial metabolism, vascular function, and inflammatory pathways. Additionally, the review examines both well-established and recently developed therapeutic approaches, such as gene treatments, pharmacological interventions, and novel biomaterial-based techniques, that are intended to mitigate hypoxia-induced cardiovascular dysfunction. By bridging fundamental molecular insights with clinical applications, this review seeks to highlight current challenges, identify critical gaps in existing knowledge, and propose future directions for advancing therapeutic strategies targeting cardiovascular complications.

Despite extensive research on intermittent hypoxia and its role in cardiovascular health, a comprehensive synthesis integrating molecular mechanisms, clinical findings, and therapeutic strategies remains limited. Most studies tend to focus on isolated aspects of IH's effects, such as its contribution to specific cardiovascular diseases or the exploration of individual therapeutic interventions. However, a holistic understanding of how IH influences cardiovascular health at the molecular, physiological, and clinical levels, particularly in the context of various therapeutic approaches, is lacking. Furthermore, while there is a growing interest in IH's potential therapeutic uses, such as in intermittent hypoxic conditioning (IHC), the full clinical implications and effectiveness of these treatments remain unclear. This review aims to bridge that gap by synthesizing existing research and providing a comprehensive analysis of the dual role of IH, both as a contributor to CVD and as a potential therapeutic tool. By examining molecular pathways, clinical data, and emerging therapies, this review seeks to offer a more integrated perspective and propose future research directions that could lead to more effective treatment strategies for hypoxia-induced cardiovascular dysfunction.

## Review

Pathophysiology of hypoxia in cardiovascular health

High Altitude

An initial cardiac response to rapid high-altitude ascent includes elevated systemic blood pressure [11], enhanced heart muscle contraction, increased cardiac output, primarily from the higher heart rate [12], despite a possible slight decrease in stroke volume, and increased heart rate because of sympathetic activation from low oxygen [13]. Acclimatization causes pulmonary artery pressure to rise, heart rate to stay high, stroke volume to fall due to fluid loss, and cardiac output to return to baseline, with a higher heart rate and lower stroke volume [14]. In healthy people, however, left ventricular (LV) function is usually preserved. People who have heart problems may find these alterations difficult [15]. There is little data on all forms of cardiac disease at higher elevations, even though low to moderate altitude usually does not exacerbate cardiovascular problems for cardiac patients [16]. Physiological knowledge indicates that some conditions call for caution.

Risks of overactivation of the adrenergic system: Patients who suffer from disorders such as tachyarrhythmias that are exacerbated by elevated adrenaline may be vulnerable at high elevations [12]. Even a moderate altitude increases the risk of PH [17]. High altitude increases the chance of conditions such as right-to-left shunt that cause low blood oxygen levels at sea level [18].

**Figure 2:**
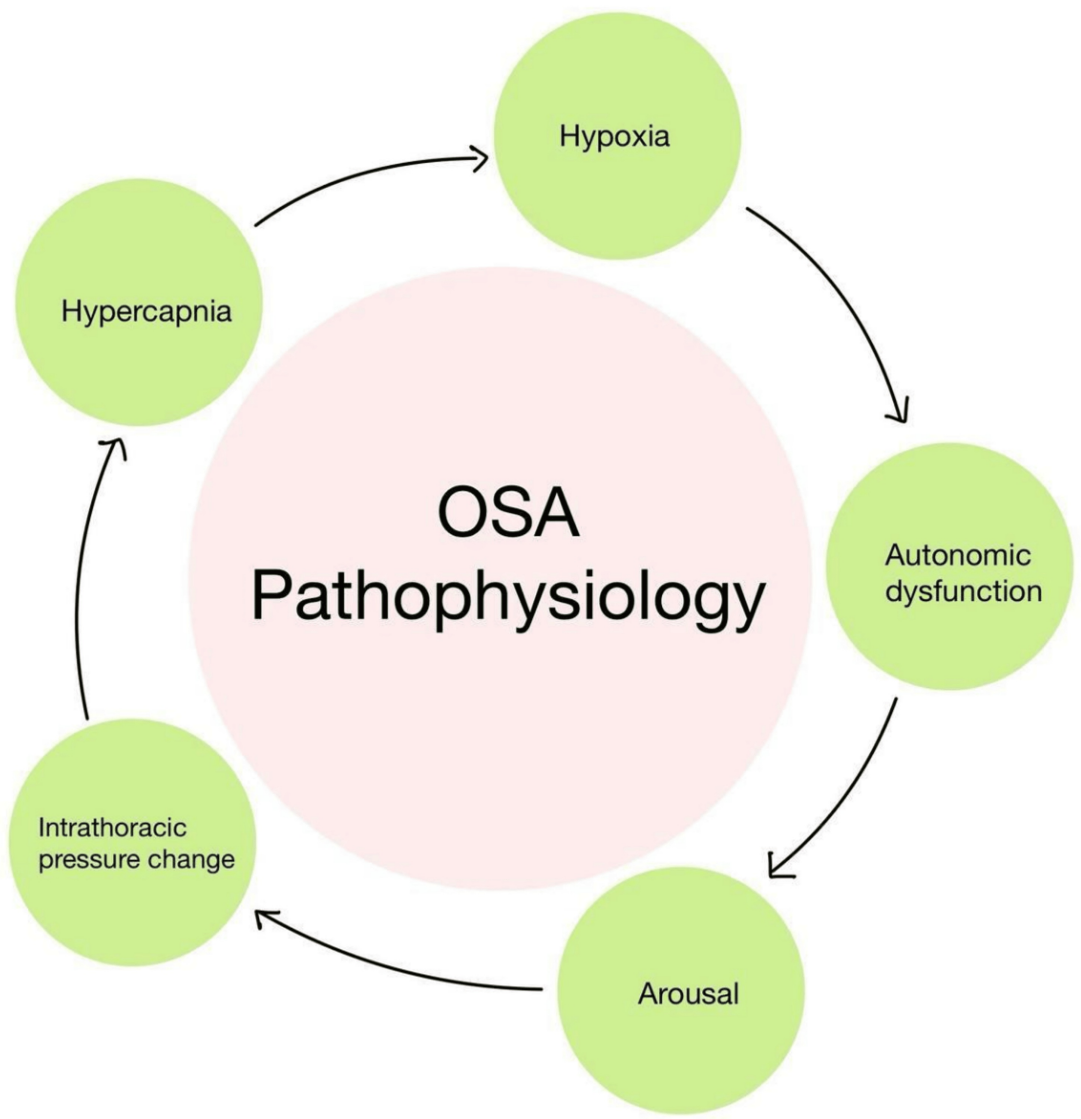
Impact of obstructive sleep apnea (OSA) and obesity hypoventilation syndrome (OHS) on cardiovascular outcomes

Coronary artery disease (CAD): During exercise, decreased oxygen availability at altitude raises heart rate and myocardial demand, which may reduce the threshold for ischemia [1]. Instead of using workload to control activity, patients should use heart rate. Moderate altitudes may be tolerated by stable, low-risk patients. It is interesting to note that the reduced incidence of stroke and coronary heart disease mortality has been linked to higher altitudes [19]. Figure 2 shows the impact of obstructive sleep apnea and obesity hypoventilation syndrome (OHS) on cardiovascular outcomes.

IH exposure at high altitude prompts a range of cardiovascular adaptations aimed at maintaining oxygen delivery and ensuring tissue perfusion under reduced atmospheric oxygen availability. Adaptation mechanisms include increased sympathetic activity, leading to elevated heart rate and blood pressure, as well as changes in ventilatory responses and vascular tone that enhance oxygen uptake and transport. At the molecular level, IH stimulates the activation of hypoxia-inducible factors (HIFs), which mediate key transcriptional responses responsible for cardiovascular remodeling and increased resilience to ischemia-reperfusion (I-R) injury. Evidence from clinical and experimental studies shows that high-altitude IH can promote myocardial protection, reduce the incidence of major adverse cardiovascular events, and improve cardiac function by enhancing autonomic cardiovascular control and upregulating protective pathways in the myocardium. However, the specific profile of IH exposure and individual variability may influence the balance between adaptive and maladaptive cardiovascular responses [20].

Pulmonary Causes

OSA and OHS: Repeated oxygen decreases brought on by OSA's periodic airway blockage result in pressure shifts, oxidative stress, and sympathetic surges. Heart failure, coronary artery disease, stroke, PH, arrhythmias (particularly atrial fibrillation, or AF), and hypertension [21] are all caused by this cascade. Inflammation and insulin resistance are examples of metabolic disorders that play a role [22,23]. These interrelated processes dramatically increase the incidence and mortality of cardiovascular disease, generating a vicious cycle in which diseases like hypertension exacerbate OSA. Chronic hypoxemia and hypercapnia brought on by obesity hypoventilation syndrome cause systemic endothelial impairment and pulmonary hypertension [24]. The risk of CVD is greatly raised by obesity-related inflammation, autonomic/metabolic dysfunction, and nocturnal stress, which all contribute to systemic hypertension, atherosclerosis, and an increased cardiac workload [25].

COPD and interstitial lung disease (ILD): Both COPD and ILD share several risk factors with CVD, especially smoking, which is a common cause of both respiratory and cardiovascular complications. The pathophysiology of COPD involves exacerbations, hyperinflation, hypoxemia, and systemic inflammation, all of which contribute to arterial stiffness and exercise intolerance, leading to cardiovascular dysfunction [26]. Specifically, in smoking-related COPD, the presence of mucus plugs and emphysema independently worsens airflow (measured by forced expiratory volume in one second, or FEV1) and oxygenation (measured by SpO_2_) [27]. Mucus plugs obstruct airways and impair ventilation-perfusion matching, leading to hypoxemia, which in turn drives systemic inflammation and oxidative stress [28]. These common mechanisms, oxidative stress, systemic inflammation, and hypoxemia, play a significant role in the development and progression of CVD in both COPD and ILD. In the context of idiopathic pulmonary fibrosis (IPF), the persistent hypoxia and lung inflammation also lead to oxidative stress and inflammation, contributing to endothelial damage. This promotes the development of atherosclerosis and plaque formation, which are characteristic of coronary artery disease [29]. Furthermore, hypoxemia increases cardiac workload, putting additional strain on the coronary arteries. While IPF shares some common risk factors with cardiovascular disease, its unique inflammatory and hypoxic environment accelerates the onset and progression of CAD, highlighting the complex interaction between these disorders [26].

**Figure 3:**
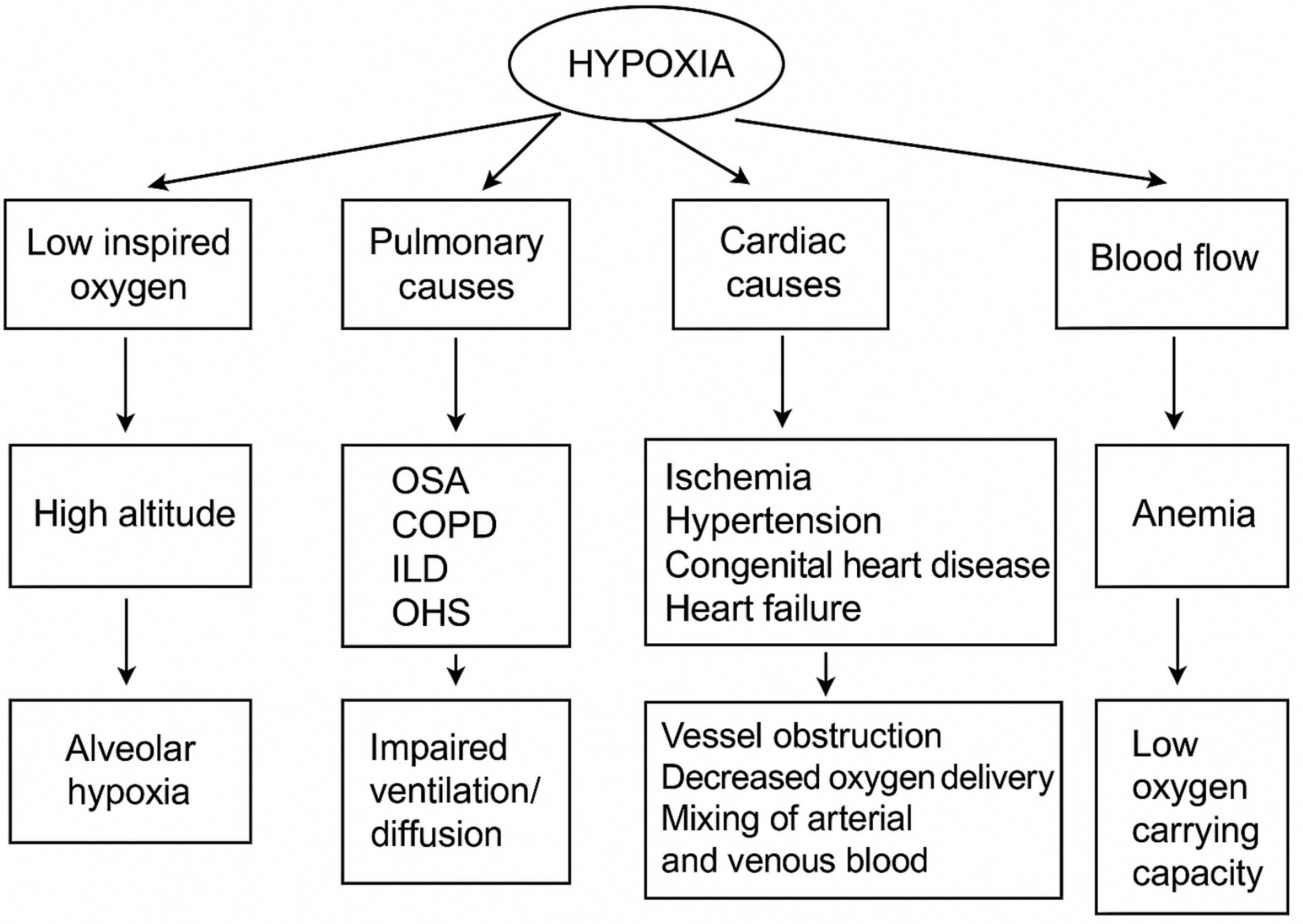
Effects of chronic intermittent hypoxia on pulmonary hypertension OHS: obesity hypoventilation syndrome; OSA: obstructive sleep apnea; COPD: chronic obstructive pulmonary disease; ILD: interstitial lung disease

PH: Through cellular alterations and signaling abnormalities, hypoxia, a contributing factor to PH, causes remodeling of the pulmonary arteries. This remodeling increases vascular resistance in all PH subtypes, leading to elevated pulmonary artery pressure and right heart strain [30]. This process is mediated by HIFs, mitochondrial dysfunction, endothelial-to-mesenchymal transition, and genetic predisposition. Endothelial dysfunction is characterized by increased endothelin-1 and reduced endothelial nitric oxide synthase (eNOS) and prostacyclin. Inflammation and oxidative stress, often linked to comorbidities, further contribute to this complex pathogenesis [31]. Figure 3 shows the effects of chronic intermittent hypoxia (CIH) on pulmonary hypertension. These cellular changes translate clinically into higher AF prevalence among OSA patients.

Cardiac Causes and Molecular Mechanisms

Atrial fibrillation: Arrhythmogenesis is caused by a complicated interaction of variables. A sensitive substrate for AF is created when local hypoxia in the atria sets off oxidative and inflammatory stress, which results in fibrosis, connexin remodeling, delayed conduction, and cell death. Another important factor is autonomic dysfunction, which is brought on by changed receptor input and increased cardiomyocyte sensitivity. Acute apneas can result in atrial fibrillation through parasympathetic activation and atrial effective refractory period (AERP) shortening. Abnormal calcium handling and atrial stretch exacerbate AF [1].

**Figure 4:**
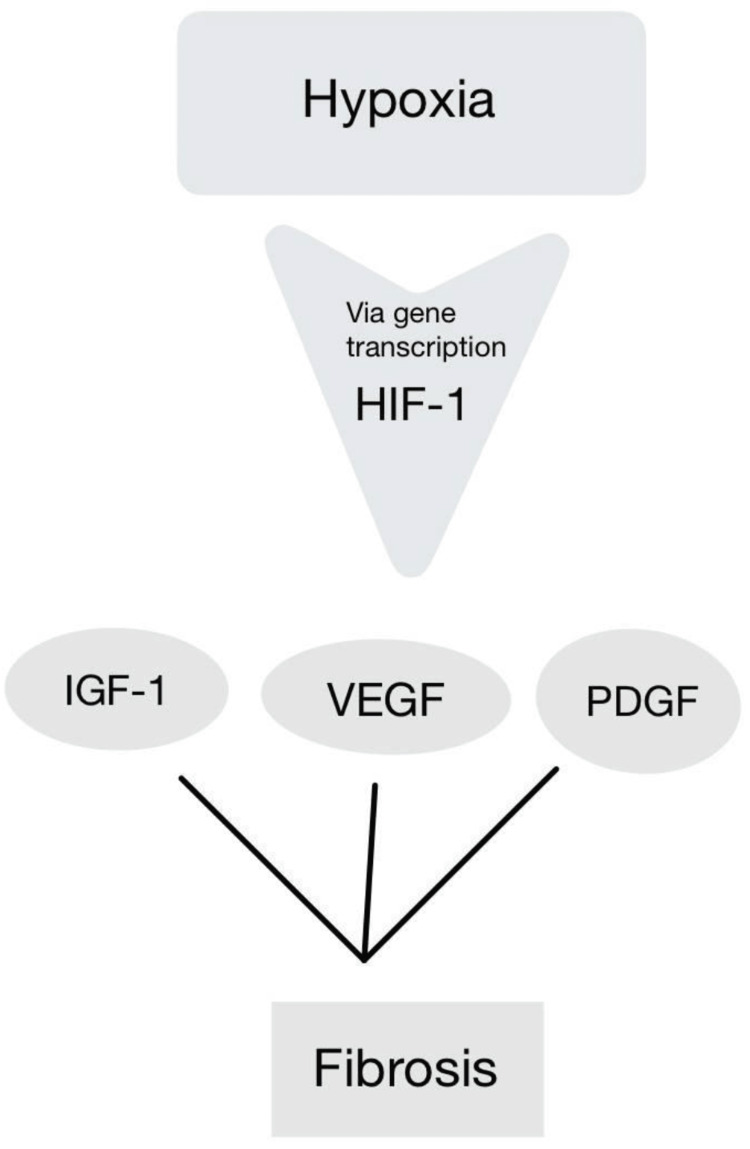
Hypoxia-induced molecular changes contributing to vascular remodeling HIF-1: hypoxia-inducible factor 1; IGF-1: insulin-like growth factor 1; VEGF: vascular endothelial growth factor; PDGF: platelet-derived growth factor

Atherosclerosis: Vascular disease is largely caused by inflammation. In rodent arteries and human endothelial cells, IH triggers nuclear factor-kappa B (NF-κB), which lowers nitric oxide (NO) and encourages atherosclerosis and intima-media thickening. Toll-like receptor-4 (TLR4) signaling [32] is essential for this NF-κB-mediated inflammation [33], which raises iNOS, monocyte chemoattractant protein-1 (MCP-1), TNF-α, and IL-6. IH also contributes to endothelial dysfunction and atherogenesis by inducing early adipose tissue inflammation. Furthermore, leukotrienes, which are increased in models of OSA and CIH, encourage early atherosclerosis, monocyte activation, and vascular remodeling [2]. Figure 4 shows a graphical representation of hypoxia-induced molecular changes contributing to vascular remodeling.

Ischemia: Damage to proteins and lipids results from vascular oxidative stress brought on by elevated ROS in different arteries caused by hypoxia. Increased activity of xanthine oxidase and NADPH oxidase combined with eNOS uncoupling causes this imbalance [34]. Superoxide dismutase and other antioxidant defenses are weakened by IH. In models of obesity and atherosclerosis, these effects intensify, encouraging fibrosis, inflammation, and altered vascular tone, which eventually results in arterial stiffness and dysfunction [35].

IH and cardiovascular disorders

Systemic Hypertension

IH significantly contributes to hypertension by influencing the sympathetic nervous system and vascular function. Preclinical studies conducted by Prabhakar et al. demonstrated that recurrent exposure to IH in rats enhanced the sensitivity of the carotid body and maintained sympathetic nervous system activity, both of which elevate blood pressure [36]. These findings have been validated by human investigations. A meta-analysis of randomized controlled trials (RCTs) evaluating CPAP treatment in OSA showed modest reductions in systolic blood pressure (approximately 2-4 mmHg). This indirectly implies that untreated OSA may cause blood pressure elevations of a similar or slightly greater magnitude. For example, in one meta‑analysis, the mean SBP reduction was −2.09 mmHg (95% CI −2.78 to −1.40) in pooled RCTs of CPAP. In human cohorts (e.g., children/adolescents followed up into adulthood), untreated moderate‑to‑severe OSA has been associated with ~6.5 mmHg higher nocturnal systolic BP compared to non‑OSA controls [37]. Fava et al. demonstrated that CPAP treatment led to modest yet significant reductions in blood pressure among patients with hypertension and OSA, reinforcing the concept that IH plays a role in the onset of hypertension [37,38].

Atherosclerosis

Atherosclerosis is a multifaceted inflammatory condition affecting major arteries. To study the various impacts of OSA-associated chronic intermittent hypoxia on atherosclerosis, the use of animal models is important. In a study, Fang et al. explored whether OSA-associated CIH independently causes atherosclerosis without the confounding effects of a high-cholesterol diet. They created two improved animal models (ApoE-KO and ApoE-p50-DKO). The models demonstrated that ApoE-p50-DKO mice had more severe disease than ApoE-KO animals, and that CIH alone can induce atherosclerotic lesions in a time-dependent manner. The study demonstrated the protective effect of NF-κB p50 in lowering vascular inflammation and CIH-induced hypercholesterolemia. Loss of p50 led to increased serum cholesterol, decreased low-density lipoprotein (LDL) receptor expression, and worsened inflammatory reactions. These models offer crucial insights into the early molecular pathways of CIH-induced atherosclerosis and more accurately depict the connection between OSA and cardiovascular disease [39]. Savransky et al. showed that chronic IH markedly enhances atherosclerotic plaque formation in ApoE-deficient mice, revealing direct mechanistic evidence of IH-induced vascular damage via inflammatory and oxidative stress pathways [40]. Polotsky et al. indicated that IH precipitates dyslipidemia in mouse models, subsequently accelerating atherosclerosis. Their research highlighted metabolic abnormalities as a significant component in IH-induced vascular pathology [40]. Preclinical models convincingly demonstrate that IH promotes atherosclerosis via inflammatory, oxidative, and metabolic mechanisms, while clinical studies have validated these findings by revealing similar pathophysiological alterations and outcomes in patients with OSA.

Drager et al. found that individuals with OSA showed elevated markers of subclinical atherosclerosis and inflammation, indicating IH as a crucial mediator of vascular damage in clinical settings [23]. Jelic et al. showed that IH in OSA patients results in endothelial damage, a precursor to atherosclerosis, predominantly mediated by oxidative stress pathways [41].

Arrhythmias

IH, a key aspect of OSA, has increasingly been associated with the onset of cardiac arrhythmias due to its effects on autonomic processes, alterations in cardiac structure, and electrical instability. Morand et al. investigated the effects of chronic IH on myocardial ischemia-related ventricular arrhythmias in rats. Studies showed that chronic IH markedly increases susceptibility to life-threatening ventricular arrhythmias during myocardial ischemia. The study revealed significant electrophysiological changes, such as prolonged QTc intervals and enhanced repolarization dispersion, which are known pro-arrhythmic substrates. These findings provide strong proof that IH can lead to heart rhythm problems, particularly in patients who already have ischemic heart disease [42]. Bober et al. found that rats exposed to IH were more likely to experience atrial arrhythmias and had noticeable problems with their autonomic nervous system. The research showed increased sympathetic tone and reduced heart rate variability as primary factors in arrhythmogenesis. These findings indicate that IH interferes with autonomic control, hence directly predisposing the heart to arrhythmias [43]. A meta-analysis showed that IH induces alterations in the heart's structure and function, including thickening of the heart muscle, scarring, and lower ejection fraction, which are all known to increase the risk of irregular heartbeats. The research also revealed a dose-response relationship, indicating that more severe or protracted IH exposure led to increased cardiac dysfunction. These findings illustrate the significance of IH as a modifiable risk factor in the development of arrhythmias, emphasizing the necessity for early treatment intervention in diseases such as OSA [44]. Park et al. investigated the therapeutic potential of IH in atrial fibrillation and found that moderate, regulated IH exposure improved cardiac electrophysiology in AF-induced mouse models [45]. IH specifically decreased sympathetic overactivity, corrected calcium-handling proteins, and reinstated the expression of connexin-43, which is essential for appropriate electrical conduction. The findings indicate that, under strictly controlled conditions, IH may unexpectedly provide beneficial anti-arrhythmic benefits, underscoring the complexity of its involvement in arrhythmia pathogenesis [45].

Takada et al. revealed a significant clinical correlation between the intensity of IH in OSA patients and the existence of low-voltage regions in the left atrium, indicative of arrhythmogenic remodeling. These structural alterations are consequential since they enhance the probability of atrial fibrillation initiation and maintenance. The results highlight that IH not only induces electrical instability but also facilitates the anatomical basis for persistent arrhythmias in human patients [46].

While preclinical studies provide strong evidence linking IH to arrhythmogenesis, including electrophysiological changes and structural cardiac alterations, several limitations should be considered. Most of the data come from animal models, which may not fully reflect the complexities of human physiology, particularly in patients with comorbidities like ischemic heart disease. Additionally, the IH exposure models in these studies vary, which could affect the generalizability of the findings to human conditions. Therefore, large prospective clinical studies are needed to confirm causality and further elucidate the role of IH in the development of arrhythmias in OSA patients, ensuring a more accurate understanding of its effects in human populations.

Heart Failure

IH has been closely linked with both the development and progression of heart failure. Preclinical studies have provided strong insights into this relationship. For example, Zhou et al. addressed the temporal dynamics of cardiac response to IH, showing that while short-term exposure activates protective antioxidant pathways, sustained exposure surpasses these defences, resulting in cardiomyopathy. These findings highlight potential therapeutic targets, including nuclear factor erythroid 2-related factor 2 (Nrf2) and metallothionein (MT), for mitigating IH-induced heart damage [47]. Additionally, a meta-analysis presented strong evidence that IH causes maladaptive cardiac remodelling in rodents, mirroring clinical findings in humans. These findings emphasize the translational significance of animal models in investigating IH-related cardiac disorders and highlight the necessity of addressing IH in clinical environments [48].

Right ventricular (RV) failure: OSA leads to RV failure through various pathways. During inspiration, negative intrathoracic pressure pushes blood into the thorax, increasing right ventricular preload [49]. Hypoxia from apnea causes hypoxic pulmonary vasoconstriction, leading to PH and elevated right ventricular afterload, which results in right heart failure over time [50]. IH may generate oxygen-free radicals, which activate inflammatory pathways, compromising vascular endothelial function and causing diminished pulmonary vasodilation. OSA also induces LV failure and exacerbates RV failure [51].

Left ventricular failure: The mechanisms contributing to LV failure in OSA are analogous to those in RV failure, being both overlapping and complex. Hypertension, renin angiotensin system (RAS) activation, obesity, IH and hypercapnia, and autonomic dysfunction contribute to LV remodelling [52]. Hypoxic pulmonary vasoconstriction leads to elevated right ventricular pressure, causing right ventricular stretching and leftward displacement of the interventricular septum, decreasing stroke volume [53]. IH and hypercapnia induce variations in sympathetic and parasympathetic activity, leading to vasoconstriction and elevated left ventricular afterload [53]. This leads to diminished stroke volume and augmented end-systolic volume, leading to maladaptive responses in the left ventricle. LV hypertrophy diminishes chamber size, leading to heart failure with preserved ejection fraction (HFpEF) and decreased ejection fraction (HFrEF) [53]. Elevated sympathetic activity stimulates the RAS system, resulting in elevated angiotensin II, aldosterone, and fluid retention, worsening heart failure. Negative intrathoracic pressure leads to enhanced atrial remodelling, elevated LV end-diastolic pressure, increased afterload, and augmented cardiac workload, escalating myocardial oxygen demand [54].

OSA as the most common cause of IH in humans

OSA affects an estimated 9%-38% of the general population, with higher rates in men (22%) compared to women (17%), and especially elevated prevalence in older adults - up to 90% in men and 78% in women [55]. OSA includes multiple subtypes, influenced by anatomical, physiological, inflammatory, and obesity-related factors. Risk for CVD in OSA patients is modulated by age, gender, symptom profile, and the extent of physiological impairment [56].

IH has gained recognition as a critical factor in driving OSA-related health consequences. The rapid, repetitive nature of IH is characterized by cycles of desaturation and reoxygenation, distinct from chronic hypoxia, and mirrors the effects of ischemia-reperfusion injury [57]. Left untreated, OSA produces systemic consequences due to a combination of disturbed sleep, intermittent changes in oxygen and carbon dioxide, negative intrathoracic pressure swings, and elevated sympathetic activation linked to disordered breathing at night.

Typical symptoms include excessive daytime drowsiness, persistent fatigue, and unrefreshing sleep [58]. The cycle of hypoxia and reoxygenation in sleep-disordered breathing (SDB) promotes inflammation and oxidative stress. These changes activate NF-κB, which in turn stimulates endothelial cells and leukocytes, upregulates adhesion molecules and VEGF, and initiates expression of hypoxia-inducible factor-1, a key regulator of oxygen homeostasis [59]. OSA diagnosis relies on detecting repeated breathing pauses during sleep lasting at least 10 seconds. As per American Academy of Sleep Medicine (AASM) guidelines (1999), an Apnea-Hypopnea Index (AHI) of five or more events per hour qualifies for diagnosis [60]. In severe instances, episodes can extend beyond three minutes or occur hundreds of times per night, frequently causing brief awakenings that disrupt sleep continuity and reduce total sleep time [61]. Without treatment, OSA raises the risk of a range of cardiovascular issues, including resistant hypertension, coronary artery disease, heart failure, arrhythmias, and stroke [62]. The pathophysiological effects of IH and fragmented sleep in OSA appear to interact with obesity and metabolic syndrome through sympathetic overactivation, oxidative stress, inflammatory responses, and neurohormonal dysregulation, supported by both clinical and experimental IH models [63].

Punjabi et al. (2009) analyzed data from a large prospective cohort of middle-aged and older adults and found a strong independent link between SDB and increased all-cause and cardiovascular mortality. This relationship was especially notable among men aged 40-70 years with severe SDB (AHI ≥30), where sleep-related hypoxemia was a key predictor of mortality, unlike arousal frequency or central apnea index [64]. In the Sleep Heart Health Study, Gottlieb et al. (2010) followed up 1,927 men and 2,495 women aged ≥40 without prior coronary heart disease (CHD) or heart failure for a median of 8.7 years [65]. OSA significantly predicted CHD incidence in men aged ≤70, with a 10% increase in CHD risk per 10-unit rise in AHI (adjusted hazard ratio (HR) 1.10; 95% CI, 1.00-1.21). Men with severe OSA (AHI ≥30) had a 68% higher CHD risk than those with AHI <5. Additionally, the risk of new-onset heart failure rose by 13% for every 10-unit AHI increase in men (adjusted HR 1.13; 95% CI, 1.02-1.26), but not in women. Severe OSA increased the heart failure risk by 58% in men [65]. Dong et al. (2013) conducted a meta-analysis of cohort studies and concluded that individuals with OSA had more than double the overall risk of developing CVD [66]. Stroke risk was significantly higher, though the association with coronary heart disease was weaker and not always statistically significant. These findings held in high-quality studies with long-term follow-up and careful risk factor adjustment [66].

Loke et al. (2012), in their systematic review and meta-analysis of nine prospective studies, found OSA to be significantly associated with an increased risk for both stroke (odds ratio (OR) 2.24; 95% CI, 1.57-3.19) and cardiovascular mortality (OR 2.09; 95% CI, 1.20-3.65). AHI severity demonstrated a dose-response relationship. Although overall association with IHD was not statistically significant (OR 1.56; 95% CI, 0.83-2.91), studies focusing on men showed a stronger link (OR 1.92; 95% CI, 1.06-3.48) [67]. Collectively, these findings reveal that OSA significantly increases cardiovascular risk, especially in middle-aged men with severe disease. The consistent associations with heart failure, stroke, and mortality emphasize the importance of early detection and intervention. Though the evidence connecting OSA to coronary artery disease is less definitive, overall data support OSA as a modifiable cardiovascular risk factor that warrants routine screening in at-risk patients.

Role of CPAP and its effects on cardiovascular outcomes

Global guidelines suggest that reducing systolic blood pressure by just 2-3 mmHg can lower stroke risk by 6%-8% and coronary disease by 4%-5% [68]. In patients with mild daytime symptoms, 12 weeks of CPAP therapy significantly reduced both 24-hour mean and diastolic blood pressure by 3.8 and 3.5 mmHg, respectively, which are benefits that are clinically important [69]. Further evidence shows that in untreated OSA, two months of auto-adjusting positive airway pressure (APAP) therapy can lower both systolic and diastolic sleep-time blood pressure, similar to fixed CPAP.

These reductions are accompanied by decreased nocturnal sympathetic activity, indicated by lower catecholamine excretion [70]. Marin et al. conducted a 10-year study including 1,651 men, finding that those with untreated severe obstructive sleep apnea-hypopnea syndrome (OSAHS) had notably higher rates of fatal (1.06 per 100 person-years) and non-fatal (2.13 per 100 person-years) cardiovascular events than healthy controls (0.30 and 0.45 per 100 person-years, respectively). CPAP-treated patients had much lower event rates (0.35 fatal and 0.64 non-fatal), similar to healthy individuals. Adjusted analysis confirmed untreated OSAHS independently increased cardiovascular risk (fatal OR 2.87; 95% CI, 1.17-7.51; non-fatal OR 3.17; 95% CI, 1.12-7.51) [71]. Martínez-García et al. performed a five-year prospective study that included 166 post-ischemic stroke patients with moderate to severe OSA. Those with AHI ≥20 who did not tolerate CPAP had a significantly higher adjusted mortality risk (HR 2.69; 95% CI, 1.32-5.61) versus patients with AHI <20. Among OSA patients, non-adherence to CPAP was linked to increased mortality (HR 1.58; 95% CI, 1.01-2.49; p = 0.04), while adherent individuals had mortality comparable to non-OSA patients [72].

Marin et al. (2005) compared cardiovascular outcomes across untreated, CPAP-treated OSA patients, and healthy controls. Their findings confirmed a higher incidence of fatal and non-fatal cardiovascular events in untreated severe OSA, while CPAP use significantly reduced those risks [71]. Finally, the SLEEP-AF (Sleep Apnoea and Atrial Fibrillation: The Effect of Treatment of Sleep Apnoea on Atrial Fibrillation Burden) study by Nalliah et al. (2022) showed that CPAP therapy in OSA patients with atrial fibrillation improved atrial conduction velocity, voltage, and reduced substrate complexity, suggesting beneficial structural and functional cardiac remodeling [73]. Together, this body of evidence strongly supports the cardiovascular protective role of CPAP in OSA, showing improvements in blood pressure, cardiac function, and long-term survival in affected individuals. Table 1 shows a comparison of cardiovascular outcomes between OSA treated with CPAP and no treatment.

**Table 1:** Comparison of cardiovascular outcomes: CPAP versus no treatment CPAP: continuous positive airway pressure; BP: blood pressure; OSA: obstructive sleep apnea; CV: cardiovascular; OR: odds ratio; HR: hazard ratio; CI: confidence interval Table created by Chaitanya Kumar Javvaji

Outcome parameter	CPAP treatment	No CPAP/untreated OSA
Blood pressure reduction (24-hour mean)	3.8 mmHg reduction	No significant change
Diastolic blood pressure reduction [69]	3.5 mmHg reduction	No significant change
Clinical significance of BP reduction [69]	Stroke risk ↓ 6%-8%, coronary disease ↓ 4%-5%	Continued elevated risk
Fatal CV events (10-year, per 100 person-years) [71]	0.35	1.06
Non-fatal CV events (10-year, per 100 person-years) [71]	0.64	2.13
Adjusted OR for fatal CV events [71]	1.0 (reference)	2.87 (95% CI, 1.17-7.51)
Adjusted OR for non-fatal CV events [71]	1.0 (reference)	3.17 (95% CI, 1.12-7.51)
Post-stroke mortality (non-adherent CPAP, HR) [72]	Comparable to controls	1.58 (95% CI, 1.01-2.49, p=0.04)

While the study by Martínez-García et al. supports the idea that CPAP treatment reduces mortality in stroke patients with OSA, especially in those who adhere to treatment, there is some controversy regarding its broader cardiovascular benefits [72].

Potential therapeutic strategies for IH

CPAP

Mechanism of action: CPAP is the first-line intervention in OSA treatment. By continuously applying positive pressure to the airway through a mask interface, this treatment works similarly to a pneumatic splint while permitting normal breathing [74]. CPAP is the primary treatment for OSA, especially in moderate-to-severe cases, as determined by the AHI, especially for a lower AHI linked to excessive daytime sleepiness (EDS) or an AHI greater than 15 events/hour [75]. Treatment with CPAP for individuals with OSA improves sleep-related quality of life and dramatically reduces blood pressure, tiredness, disease severity, and the risk of auto accidents [75]. OSA patients who experience EDS and marked daytime functional impairment tend to show better adherence to CPAP therapy, whereas those with comorbid conditions, milder symptoms, or female patients typically demonstrate lower adherence rates [76]. CPAP withdrawal has also been linked to a health/safety risk behavior, underscoring the complexity of variables in various domains that influence patients' acceptance of CPAP [77]. The Randomized Intervention with CPAP in CAD and OSA (RICCADSA) study found that individuals with OSA with a higher heart rate response to apneas or hypopneas are more likely to experience cardiovascular events and benefit more from CPAP therapy compared to those with a lower heart rate response [56]. CPAP was predicted to reduce cardiovascular risk by 59% in individuals with a high heart rate response, but not significantly in all comers [20].

The Sleep Apnea Cardiovascular Endpoints (SAVE) research trial showed that while CPAP does not enhance cardiovascular outcomes, it helps alleviate mild to moderately severe OSA symptoms [78,79]. However, the SAVE trial (2016) showed limited improvement in cardiovascular outcomes despite symptom relief from CPAP, suggesting that while CPAP may help manage symptoms like daytime sleepiness, its impact on long-term cardiovascular events, including mortality, remains unclear. This highlights the need for further research to better understand the role of CPAP in reducing cardiovascular risk, particularly in patients with coexisting conditions like ischemic stroke. There was no difference found in cardiovascular outcomes between the treatment and control groups, which included 2,717 people with moderate or severe OSA over the years, even though there was a significant decrease in daytime drowsiness and there were fewer cases of restricted breathing when sleeping [79]. CPAP is without a doubt the gold standard for treating OSA, but it does not address the pathophysiological mechanisms governing its pathogenesis, particularly in overweight and obese individuals, and treatment adherence is frequently suboptimal (up to 80% of individuals with OSA fail to adhere) [80]. OSA management is limited due to focusing on symptoms rather than the underlying disease, and adherence to therapies is variable. Nasal PAP is the preferred treatment, but its benefits for cardiometabolic outcomes are not strongly supported. Alternative therapies like mandibular advancement therapy and hypoglossal nerve stimulation are supported with limited clinical evidence. Thus, alternative options for the treatment of OSA are needed to yield better long-term outcomes [79,81]. High-quality randomized controlled trials support CPAP as the first-line therapy for OSA, though evidence for its benefit in cardiovascular outcomes remains inconclusive, and long-term adherence is a significant challenge.

Behavioural therapy and lifestyle modifications

Weight Reduction

For overweight patients with OSA, weight loss is the most crucial objective because the start and progression of OSA are directly correlated with greater neck fat distribution. The AASM advises individuals who are overweight to aim for a BMI of 25 kg/m^2^ [82]. A randomized controlled trial found that losing weight reduced the severity of OSA symptoms and that patients also experienced improvements in blood glucose, cholesterol, and inflammatory biomarkers [78]. Furthermore, in obese people with metabolic syndrome, it has been shown that individuals at high risk of OSA tend to lose less weight following dietary counseling compared to those at low risk [83]. Exercise dramatically reduces the cardiovascular issues linked to OSA. Physical exercise is advised for patients with OSA. The Action for Health in Diabetes (AHEAD) study found that lifestyle changes like exercise positively impact OSA; 10-year follow-up polysomnograms showed that weight loss through intensive lifestyle intervention reduced OSA severity [84].

Alcohol Avoidance

A systematic review and meta-analysis of 21 studies from 1985 to 2015 found that higher alcohol consumption increases the risk of OSA by 25% when compared to those who do not consume alcohol [85]. The effects were attributed to the hypoglossal nerve or a selective adverse effect on genioglossus muscle activity and airway dilator muscles [74]. Weight loss and exercise are key behavioral modifications that show moderate to strong evidence in improving OSA severity, though the effectiveness can vary depending on individual patient factors such as metabolic risk. Evidence supports the avoidance of alcohol as a preventive strategy for OSA, with moderate evidence linking alcohol consumption to an increased risk of OSA episodes.

Oral Advancement Devices (OADs)

OADs aim to improve pharyngeal airway space and reduce airway collapse by realigning oral or craniofacial structures. Mandibular advancement devices (MADs) widen and stabilize the upper airway by moving the jaw forward, while also impairing upper airway flow during sleep [86]. Patients with mild-to-moderate OSA and those with severe OSA can benefit the most from MADs. MADs widen the upper airway and make it less collapsible as one sleeps, by moving the tongue and jaw anteriorly [87]. These are recommended in patients who cannot tolerate CPAP [58]. Here, therapeutic adherence is higher compared to CPAP, even though the AHI drop linked to MADs is sometimes insufficient. Patients often say that MADs are more comfortable, quiet, and transportable than CPAP devices [88]. A meta-analysis of individual patients revealed that both devices had comparable outcomes for significant subjective variables like quality of life and daytime sleepiness. With increases in both N3 and rapid eye movement (REM) sleep, polysomnography (PSG) demonstrated that both therapies enhanced sleep architecture [89]. The use of MADs did not affect blood pressure, endothelial cell function, or inflammatory indicators, according to randomized controlled trials [90].

Nonetheless, following MAD treatment, some research showed beneficial cardiovascular outcomes, including a reversal of left ventricular remodeling [91]. Usually, the best kind of treatment is custom titratable MADs. The lower and upper teeth are covered by these two-piece devices, which enable titration, a gradual alteration of the mandible by incremental advancements [92]. Young age, female gender, no obesity, small neck circumference, and mild-to-moderate AHI are predictors of a positive response to a MAD, with retraction of maxilla and mandible, narrow airway, and short soft palate [91]. A poor MAD response is predicted by high upper airway collapsibility, defined as a CPAP therapeutic pressure exceeding 10.5 cmH_2_O. Recent studies have revealed that patients with low MAD responsiveness often experience significant ventilatory instability during sleep or a significant loop gain [91]. Several limitations limit the use of MADs in therapeutic settings.

Dental conditions such as an inadequate maximal protrusive distance, tooth loss, and temporomandibular joint pain exclude MADs as a possible treatment for more than one-third of patients [93]. To prevent dental adverse effects like tooth movements or bite alterations, follow-ups are usually necessary every six months. If the mandible is moved too far forward, the lateral diameter of the airway may decrease, decreasing its effectiveness. Furthermore, the precise clinical traits that point to MADs as a successful treatment have not yet been identified [94]. Mandibular advancement devices are cost-effective and clinically effective for mild-to-moderate OSAH, improving effectiveness estimates and reducing health economic model uncertainty [95]. To evaluate the impact of MADs on cardiovascular, respiratory, and other types of mortality, long-term interventional randomized trials are required. MADs provide a valuable alternative to CPAP for mild-to-moderate OSA with high patient adherence, though the effectiveness in reducing AHI and improving cardiovascular outcomes remains less robust when compared to CPAP.

CPAP vs. MAD

In a study, patients with polysomnography-diagnosed OSA were divided into two groups: one received eight weeks of CPAP treatment, while the other group received a custom-made, two-piece, adjustable MAD. CPAP patients showed improved short-term PSG results, clinical symptoms, and quality of life, even in severe OSA patients, with similar improvements with CPAP and MAD after drug-induced sleep endoscopy (DISE) evaluation [96]. A May 2021 database search of 436 studies found that CPAP significantly outperformed MAD in reducing AHI and raising oxygen saturation levels, but no significant difference was found in Epworth Sleepiness Scale (ESS) scores between the two treatments [97].

Mid-frequency Anti-snoring Devices

The device, typically worn in the submandibular region, alerts a patient to stop snoring when they sleep in a supine position. A study involving 50 patients found that a mid-frequency anti-snoring device effectively reduced snoring length, AHI episodes, and duration of SpO_2_ below 90% in moderate-to-severe OSA patients [98].

Upper airway surgery

Patients with OSA should only be offered surgical alternatives if conservative therapies and airway devices have not been effective.

Uvulopalatopharyngoplasty

The goal of upper airway surgery is to prevent pharyngeal collapse by improving anatomy. Uvulopalatopharyngoplasty (UPPP) is the most common type of upper airway surgery for OSA. UPPP, a procedure that removes the uvula and soft palate, reduces pharyngeal collapse and increases the retropalatal lumen volume, often accompanied by a tonsillectomy [88]. With an average difference of 18.59, UPPP dramatically decreased the average AHI difference for adult OSA patients after surgery. Additionally, it considerably reduces daytime sleepiness as measured by the ESS [93]. A meta-analysis of 15 observational studies reported a 33% overall reduction in apnea-hypopnea index risk, while the same analysis found that laser-assisted uvulopalatopharyngoplasty alone reduced AHI risk by only 17% [99].

However, UPPP usually results in velopharyngeal insufficiency, soft palatal edema, nasopharyngeal regurgitation, rhinolalia, dysphagia, and aberrant scarring with velopharyngeal stenosis [100]. Palatal surgery has been extended to include techniques that do not involve complete ablation. Lateral sleeping position therapy is gaining popularity as a treatment option for obstructive sleep apnea. If a patient has a hypertrophic tongue, UPPP may be used with radiofrequency thermotherapy (RFTT) [101]. Furthermore, it has been demonstrated that maxillo-mandibular advancement (MMA) surgery is a successful treatment, with 80% of patients experiencing an immediate improvement. Patients with a higher AHI, a lower BMI, and a younger age are more likely to benefit from MMA [102,103]. Other palate procedures like barbed reposition pharyngoplasty (BRP) and expansion sphincter pharyngoplasty (ESP) provide a possibly safer and more effective approach for treating pharyngeal obstruction. UPPP shows moderate effectiveness in improving AHI and daytime sleepiness but comes with significant surgical risks and a limited effect on long-term OSA control.

Tongue Reduction Surgery

An additional procedure to uvulopalatopharyngoplasty has been suggested: midline glossectomy, which entails excising the dorsal surface of the tongue's elliptical tissue [104]. A trial involving 45 patients with moderate-to-severe OSA found that AHI was reduced by over 50% in 75% of patients undergoing transoral robotic surgery and 62.1% of patients undergoing tongue base coblation resection. Postoperative bleeding, foreign body sensation, and taste perception impairment are less common in patients following transoral robotic surgery (TORS) [105].

Hypoglossal Nerve Stimulation (HSN)

In 2014, HSN was presented as a therapy for OSA. The hypoglossal nerve contains motor fibers that innervate several muscles, including the primary pharyngeal dilator muscle, the genioglossus [91]. A hypoglossal nerve stimulator, similar to a pacemaker, stimulates the tongue to move away from the airway, preventing airway collapse while breathing [78]. A study investigated the impact of hypoglossal nerve stimulation on OSA; AHI, patients' sleepiness levels, sleep quality, snoring prevalence, and other polysomnography measurements were also monitored. During the three-year follow-up, the median AHI incidents decreased from 28.2 events/hour to 6.2 events/hour [78]. Adult patients with moderate-to-severe OSA, no more than 25% of central or mixed events, BMI under 35 kg/m^2^, and absence of complete concentric palatal collapse at DISE are eligible for HSN [106]. The device implant necessitates surgical intervention by an ENT specialist, resulting in high patient adherence and satisfaction, with consistent and encouraging results over time [91]. Investigations are ongoing to understand the pathophysiological consequences and results of HSN, with efforts to determine predictors of a positive response.

Positional therapy

Positional OSA (POSA) is defined as having an AHI in the supine position that is at least twice as high as that in other positions. According to this criterion, POSA affects over half of OSA patients [107]. Sleeping in a supine position reduces the inspiratory volume, alters the shape of the airway, and restricts the use of the muscles that expand the airway [80]. AASM advises patients to avoid supine sleeping by using a pillow or bag, while the "tennis ball" technique involves strapping a bulky item to the patient's back [80]. Less than 10% of patients continue to utilize this treatment 30 months after prescription, despite its proven benefits in reducing sleep time spent in supine positions, so it is less effective [108]. Emerging positional therapy options include neck or chest devices that can adjust the supine sleeping posture by stimulating the body with a subtle vibration alarm [109]. Patients who suffer from shoulder issues or any other physical impairment that prevents them from sleeping in the lateral position are not advised to use this technique [88]. Although it is not suitable for all OSA patients, positional treatment works better for younger and less obese persons [110]. There is currently no information on any complications over time or the long-term compliance of these novel therapies.

Pharmacological therapies

As of right now, the Food and Drug Administration (FDA) has not approved any pharmacological medications that are widely used to treat OSA. Pharmaceuticals that have FDA approval are currently only used to treat symptoms, not illnesses. Antidepressants like fluoxetine are pharmacological therapies used to enhance the patency of upper airway muscles during sleep. Selective serotonin reuptake inhibitors (SSRIs) have been investigated as a treatment for OSA since sleep-dependent serotonin availability is what stimulates upper airway dilator motor neurons [88]. Hanzel et al. reported that patients receiving fluoxetine experienced a mean AHI drop from 57 to 34 events per hour [110]. According to studies, protriptyline and fluoxetine decreased rapid eye movement sleep, which in turn decreased the frequency of apnea and hypopnea episodes [104].

A prospective crossover unblinded trial on 12 patients found that fluoxetine was better tolerated than protriptyline, with six patients showing good responses to either medication [104]. The risk of occupational accidents is doubled for patients with OSA-related daytime sleepiness, and one of the main causes of highway accidents and fatalities is driver sleepiness. Modafinil and armodafinil are central nervous system (CNS) stimulants that can promote daytime wakefulness in this population [78]. Modafinil, armodafinil, and solriamfetol are FDA-approved medications for treating excessive sleepiness associated with OSA, believed to improve wakefulness by inhibiting dopamine and norepinephrine reuptake [111].

Topiramate, acetazolamide, and zonisamide are examples of carbonic anhydrase inhibitors that lessen the adverse effects on AHI [104]. The obstruction of the airway caused by allergic rhinitis might make OSA worse. Nasal corticosteroids, specifically fluticasone, are believed to widen the upper airway, potentially reducing symptoms of OSA [78]. In a study of 11 participants who completed three overnight in-laboratory sleep studies, data from 10 individuals showed that the combination of atomoxetine with solifenacin and biperiden improved upper airway function and reduced sleepiness in OSA patients, though the effects were less pronounced compared to the combination of atomoxetine with oxybutynin. New research indicates that optimal combination pharmacotherapy strategies for treating OSA should involve broad antimuscarinic M-subtype receptor selectivity or possibly M2 muscarinic receptor selectivity with high blood-brain barrier permeability [112]. Pharmacological therapies for OSA, while approved for symptom management, lack robust evidence for long-term effectiveness in controlling the disorder itself, and further research is needed to confirm their role in treatment.

Rehabilitation therapies

Myofunctional therapy when used in conjunction with CPAP has been proven to enhance the tone of oropharyngeal muscles and other soft tissues in the upper airway. Myofunctional treatment targets the tongue and pharyngeal muscles using isotonic and isometric oropharyngeal exercises. Patients with mouth breathing and nasal blockage have demonstrated the efficacy of functional nasal breathing rehabilitation approaches. These patients apparently slept better, snored less, and mouth-breathed less [113]. Intermittent hypoxic-normoxic training (IHNT) and intermittent hypoxic-hyperoxic training (IHHT) are noninvasive methods that involve repeated exposure to a gas mixture with oxygen deficiency, followed by intervals of normoxic or hyperoxic gas mixtures.

The IHNT/IHHT technique enhances mitochondrial metabolism, prevents excess ROS generation, and stimulates endothelial nitric oxide for vasodilation and endothelial proliferation [113]. In a study, IHNT/IHHT significantly reduced heart rate, total serum cholesterol, and LDL, but did not significantly affect hematological parameters like hemoglobin, erythrocytes, and reticulocytes. The IHC group experienced an increase in peak oxygen consumption and exercise time to fatigue after the hypoxic conditioning course, but no noticeable change in oxygen uptake (VO_2_) peak was observed. However, the study had limitations due to large heterogeneity, small sample size, inability to perform meta-regression analysis, and lack of data on adverse effects and IHC treatment safety [113]. Myofunctional therapy shows promising results when combined with CPAP, particularly for improving sleep quality and reducing snoring, though more research is needed to standardize its use in clinical practice.

Importance of early recognition and treatment of IH

Early recognition and timely treatment of IH are crucial to preventing the progressive cardiovascular damage that often precedes overt disease. Even in asymptomatic individuals, IH commonly arising from conditions like OSA or central sleep apnea can silently initiate physiological stress, including nocturnal hypertension, left ventricular remodeling, endothelial dysfunction, and metabolic disturbances, which over time can culminate in serious outcomes such as heart failure, atrial fibrillation, or myocardial infarction if left untreated [1,114-116]. Systematic screening for IH in high-risk populations, including those with resistant hypertension, diabetes, obesity, metabolic syndrome, or previous cerebrovascular events, is essential to interrupt disease progression before irreversible structural damage occurs [1,114-116]. Tools like overnight oximetry and PSG help identify even minimal nocturnal oxygen desaturation, which substantially elevates cardiovascular risk [115,117].

Additionally, early recognition of IH-related metabolic disturbances, such as hepatic de novo lipogenesis linking IH to nonalcoholic fatty liver disease, further underscores the broader systemic impact of untreated hypoxia [118]. In occupational settings, like high-altitude mining, where chronic IH and disrupted sleep patterns are common, early detection and targeted interventions improve both cardiovascular outcomes and the overall well-being of workers [119]. Emerging research highlights that prompt therapeutic intervention may not only halt but even reverse early pathological changes [120]. Molecular evidence shows that early vascular changes, such as decreased nitric oxide availability and increased endothelin-1, can precede overt cardiovascular disease, making early biomarker-based screening a promising strategy [121-123]. Particularly in vulnerable populations, including obese adolescents and sedentary individuals, proactive treatment of IH through CPAP, weight management, or surgical options can significantly lower long-term cardiovascular and metabolic risks [117,124]. Integrating sleep health assessment into cardiovascular preventive strategies is therefore indispensable for preserving both metabolic stability and cardiovascular integrity.

On the other hand, recognizing the critical window for early intervention has led to growing interest in utilizing IH as a therapeutic tool under controlled conditions. The timing of such interventions, particularly IH conditioning (IHC), is vital. Animal studies reveal that properly timed hypoxic exposure post-myocardial infarction can reduce scar formation and enhance ventricular function, whereas delayed interventions lose efficacy, highlighting a narrow therapeutic window that must be utilized for maximal benefit [15,120]. This represents the other end of the spectrum, where controlled IH may be employed for cardiovascular benefit, in contrast to the harmful, unregulated forms associated with sleep-disordered breathing.

Future directions

To translate current knowledge into improved cardiovascular outcomes, future research should pursue the following key directions.

Advancing Personalized Medicine Approaches

Inter-individual variability in IH responses necessitates the adoption of personalized medicine strategies. Phenotyping patients based on physiological traits such as loop gain, upper airway collapsibility, arousal threshold, and fluid redistribution may help tailor treatments like CPAP, adaptive servo-ventilation (ASV), or nerve stimulation [116]. Genetic factors influencing HIF signaling or antioxidant capacity could also inform risk stratification and treatment responsiveness [19]. Table 2 shows key biomarkers for personalized sleep apnea medicine.

**Table 2:** Key biomarkers for personalized sleep apnea medicine CRP: C-reactive protein; IL-6: interleukin 6; TNF-α: tumor necrosis factor-alpha; 8-OHdG: 8-hydroxy-2'-deoxyguanosine; TBARS: thiobarbituric acid reactive substances; microRNA: microribonucleic acid; E-selectin SNP: endothelial selectin gene single nucleotide polymorphism; Pcrit: pharyngeal critical closing pressure; ASV: adaptive servo-ventilation Table created by Chaitanya Kumar Javvaji

Biomarker type	Example biomarkers	Clinical use
Physiological endotypes [125]	Loop gain, Pcrit, arousal threshold	CPAP/ASV/HGNS response prediction
Inflammatory markers [126,127]	CRP, IL-6, TNF-α	CV risk, prognosis, subgrouping
Oxidative stress markers [127,128]	8-OHdG, TBARS	Risk stratification, therapy adjunct
Genetic/proteomic markers [125]	microRNA, E-selectin SNP	Treatment/personal risk prediction

Unraveling Mechanistic Pathways

Further elucidation of the molecular and cellular pathways involved in IH-induced cardiovascular effects is essential. In particular, the feed-forward loop between ROS and HIF-1α activation represents a potential therapeutic target [9]. Understanding the role of transcriptional and post-transcriptional regulators (e.g., miRNAs, DNA methylation) could yield novel interventions to mitigate long-term cardiovascular damage [115,119].

Optimizing Experimental and Animal Models

Animal models of CIH should be refined to more closely replicate the IH patterns and comorbidities seen in human OSA [122]. Sex- and age-specific differences must be incorporated to increase translational relevance. Longitudinal models assessing the trajectory from early IH exposure to overt cardiovascular disease are also needed.

Validating IH-Based Therapies

IH conditioning (IHC) shows promise as a cardioprotective intervention. Clinical trials should determine the optimal "dose" of IH (i.e., severity, frequency, duration) for specific conditions and populations [15,129]. Studies must also evaluate the long-term sustainability and safety of such protocols, particularly in patients with preexisting cardiovascular disease.

Expanding Biomarker Discovery

Reliable biomarkers for IH severity and systemic impact are urgently needed. Current reliance on the oxygen desaturation index or AHI fails to capture the complexity of IH exposure [24]. Integrating molecular markers such as circulating miRNAs, inflammatory cytokines, or oxidative stress indicators could improve early detection and monitoring of cardiovascular risk.

Improving Treatment Adherence and Efficacy

The inconsistent outcomes of CPAP therapy in randomized trials are often attributed to poor adherence or inappropriate patient selection [115]. Research should focus on behavioral interventions, wearable feedback technologies, and simplified delivery systems to improve adherence. Comparative studies are also needed to assess CPAP versus alternative therapies such as oral appliances, oxygen therapy, or phrenic nerve stimulation in diverse patient groups.

Investigating Special and Vulnerable Populations

More studies should include underrepresented populations, including women, elderly patients, and those from diverse ethnic backgrounds. Particular attention should be paid to patients with HFpEF, arrhythmias, or those living at high altitudes, where IH may have distinct pathophysiological effects [19,115].

Integrating Multi-modal Interventions

Future research should explore the combined effects of IH with lifestyle and pharmacological interventions. Exercise training, dietary modification, and antioxidant therapy may synergize with IH to enhance cardiometabolic resilience [17,118]. Combination protocols should be assessed in well-designed clinical trials.

Longitudinal, Real-World Data Collection

There is a need for long-term, prospective studies using comprehensive monitoring (e.g., polysomnography, actigraphy, biomarkers) to understand how different forms of IH influence cardiovascular trajectories over time. Data from occupational settings, such as miners exposed to chronic intermittent hypobaric hypoxia, may offer unique insights [119].

## Conclusions

Intermittent hypoxia presents a complex challenge to human health, with its effects depending on factors like severity, duration, and individual health conditions. While mild, controlled IH can offer therapeutic benefits such as promoting angiogenesis and enhancing oxygen utilization, severe IH, as seen in conditions like OSA, COPD, and high-altitude exposure, contributes to cardiovascular disease through mechanisms like oxidative stress, endothelial dysfunction, and metabolic dysregulation. Given these risks, timely recognition and management of IH are crucial, particularly in high-risk populations. In clinical practice, screening for OSA in cardiology settings could improve early detection and help prevent the cardiovascular complications associated with untreated sleep-disordered breathing. Treatment approaches such as CPAP, oral appliances, and even emerging therapies like intermittent hypoxic conditioning should be tailored to individual patient needs, especially for those who are intolerant to CPAP. Future efforts should focus on refining translational IH models and validating these interventions in clinical and occupational settings, integrating sleep health as a key component of cardiovascular care, and developing biomarker-driven strategies for early diagnosis and intervention. This will be vital in addressing the global burden of cardiovascular diseases related to sleep disorders.
